# A long-forgotten ‘dinosaur’ bone from a museum cabinet, uncovered to be a Japan's iconic extinct mammal, *Paleoparadoxia* (Desmostylia, Mammalia)

**DOI:** 10.1098/rsos.172441

**Published:** 2018-07-25

**Authors:** Kumiko Matsui, Yuri Kimura, Mitsuhiro Nagata, Hiroaki Inose, Kazuya Ikeda, Brian Lee Beatty, Hideyuki Obayashi, Takafumi Hirata, Shigeru Otoh, Tatsuya Shinmura, Sachiko Agematsu, Katsuo Sashida

**Affiliations:** 1 Kyushu University Museum, 6-10-1 Hakozaki, Higashi-ku, Fukuoka City, 812-8581, Japan; 2 University Museum, the University of Tokyo, 7 Chome-3-1 Hongo, Bunkyō, Tokyo 113-0033, Japan; 3 Department of Geology and Paleontology, National Museum of Nature and Science, 4-1-1 Amakubo, Tsukuba, Ibaraki 305-0005, Japan; 4 Graduate School of Science and Engineering for Education, University of Toyama, Toyama 930-8555, Japan; 5 Fukushima Museum, Aizu-wakamatsu, Fukushima 965-0807, Japan; 6 Tsuchiyu-Onsen Tourism Association, Tsuchiyu Onsen Town, Fukushima 960-2157, Japan; 7 Department of Anatomy, New York Institute of Technology College of Osteopathic Medicine, Northern Boulevard, Old Westbury, NY 11568, USA; 8 Graduate School of Sciences, Kyoto University, Sakyo, Kyoto 606-8502, Japan; 9 Geochemical Research Center, The University of Tokyo, Bunkyo, Tokyo 113-0033, Japan; 10 Graduate School of Science and Engineering for Research, University of Toyama, Toyama 930-8555, Japan; 11 Ashoro Museum of Paleontology, Ashoro, Hokkaido 089-3727, Japan; 12 Graduate School of Life and Environmental Sciences, University of Tsukuba, Tsukuba, Ibaraki 305-8572, Japan

**Keywords:** Desmostylia, *Paleoparadoxia*, femur, Miocene, Tsuchiyu, dinosaur

## Abstract

Here, we report a new ‘discovery’ of a desmostylian fossil in the geological collection at a national university in Japan. This fossil was unearthed over 60 years ago and donated to the university. Owing to the original hand-written note kept with the fossil in combination with interview investigation, we were able to reach two equally possible fossil sites in the town of Tsuchiyu Onsen, Fukushima. Through the interviews, we learned that the fossil was discovered during construction of a debris flow barrier and that it was recognized as a ‘dinosaur’ bone among the locals and displayed in the Village Hall before/until the town experienced a fire disaster in 1954. As scientific findings, the fossil was identified to be a right femur of *Paleoparadoxia* (Desmostylia), which shows well-preserved muscle scars on the surface. The age was estimated to be 15.9 Ma or younger in zircon-dating. This study shows an excellent case that historical and scientific significances could be extracted from long-forgotten uncatalogued specimens as long as the original information is retained with the specimens.

## 1. Introduction

Scientific ‘treasures’ are housed in museum collections with many specimens waiting to be catalogued and described. These specimens sometimes attract the attention of museum scientists or visiting researchers who provide new insights for evolutionary history, taxonomy and palaeoecology. Similar discoveries have been made for marine mammals as well. Tsai *et al*. [1] reported the unexpected discovery of the first pygmy right whale fossil from the Northern Hemisphere based on a collection of the Smithsonian National Museum of Natural History. This specimen was collected from Okinawa, Japan in 1948 by the US Geological Survey and has been housed in the museum. Unfortunately, the exact locality was not properly recorded in the original finding of the fossil despite that it is located somewhere in the United States Marine Corps Base Camp. They are only a few examples that evidence the value of museum collections, which could lead to unexpected discoveries. The label information is critically important for these discoveries as fossil materials cannot be fully used in research if locality information is lost. Here we report that a long-forgotten desmostylian fossil was uncovered from a museum cabinet, and that two equally possible locations were fortunately marked as the fossil locality based on the original hand-written note associated with the fossil and interview investigation.

In the spring of 2017, one of the authors (Y.K.) happened to find an old wooden box in the geological collection room at the University of Tsukuba. A desmostylian femur was in the box with an old label that contains a person's name, the date and a local address. According to the label information, this uncatalogued specimen was supposed to be discovered from the right bank of the Higashi Karasugawa River, Tategoshi, Tsuchiyu Onsen Town, Fukushima City in 1955. Despite that the local address (Tategoshi) is no longer used as the official postal address, the town, Tsuchiyu Onsen, still exists. In order to find the exact locality of the fossil and search for possible additional specimens, we interviewed local people and reviewed archived documents and photos along with the geological fieldwork. Based on comparative studies, we confirm that the fossil specimen (EESUT-PV-0001) found from Tsuchiyu Onsen town, which was known as a ‘dinosaur’ bone by locals, is desmostylian *Paleoparadoxia* and provide emended diagnosis of femoral morphologies for the Desmostylia. Desmostylia is a fossil group of Japanese iconic mammals, and a lot of skeletons and postcranial materials of the genus have been reported from the early to middle Miocene (e.g. [2]) in North Pacific Rim. In the northern part of Japan, many paleoparadoxiid specimens were reported because Miocene strata are well-exposed (Yanagawa [3]; *Desmostylus japonicus*; Iwaki [4,5]). This specimen shows the best-preserved femoral surface of all *Paleoparadoxia* specimens.

## 2. Material and methods

We compare EESUT-PV-0001 to all desmostylian genera for which the femur is known (*Behemotops*, *Paleoparadoxia*, *Neoparadoxia*, *Ashoroa*, *Cornwallius*, *Desmostylus*); observed specimens are listed below. The phylogenetic relationship of the Desmostylia to the closest living group is still under debate. Some researchers consider it to be a tethytherian (Afrotheria; [6,7]), and others included it in the Perissodactyla (Laurasiatheria; [8,9]). Thus, we include Grevy's zebra (*Equus grevyi*: NMMT-M43075), tapir (*Tapirus indicus*: M43531) and elephant (*Elephas maximus*: M52875) as potential outgroups of desmostylia for comparative studies.

### 2.1. Comparative specimens and measurements

#### 2.1.1. Measurements

The measurements of the femora were made following Inuzuka [10,11]. Measurements were made in mm units by using a digital caliper (SHINWA Digital Vernier Caliper with Hold Function 19974) and ImageJ2 (Fiji) [12].

#### 2.1.2. Institutional abbreviations

AMP: Ashoro Museum of Paleontology, Hokkaido, Japan; EESUT: Earth Evolution Science, University of Tsukuba, Ibaraki, Japan; LACM: Los Angeles County Museum, Los Angeles, CA, USA; NMNS, NSMT: National Museum of Nature and Science, Tokyo, Japan; UCMP: University of California Museum of Paleontology, Berkeley, California, USA; UHR: Hokkaido University Museum, Sapporo, Japan; USNM: Department of Paleobiology, U.S. National Museum of Natural History, Smithsonian Institution, Washington, D.C., USA.

#### 2.1.3. Comparative specimens

The following specimens and references were used in this study.

##### 2.1.3.1. Desmostylia

###### 2.1.3.1.1. *Behemotops*.

AMP 22, complete left femur of *Behemotops katsuiei* from the late Oligocene Morawan Formation, Hokkaido, Japan, described by Inuzuka [13,14]. This specimen is the holotype of *B. katsuiei*. AMP 22 has an erupted M2 and is considered as an adult.

###### 2.1.3.1.2. *Paleoparadoxia*.

NMNS PV-5601, an incomplete right and left femur of *Paleoparadoxia tabatai* [15] from the early Miocene Mizunami Group, Gifu, Japan, designated as the neotype of this species by Shikama [16]. NMNS PV-5601 shows epiphyseal fusions in the humerus and is considered as an adult.

###### 2.1.3.1.3. *Neoparadoxia*.

Two femora of *Neoparadoxia* were used for this study. One is the femur of *Neoparadoxia repeninngi* (UCMP 81302), a complete left femur from the middle Miocene Ladera Formation in California, USA. Epiphyses of the whole skeleton of this specimen were fused and the specimen is considered as an adult. The other femur is *Neoparadoxia cecilialina* (LACM 150150), a nearly complete left femur from the lower upper Miocene Monterey Formation in California, USA. Epiphyses of the femur of LACM 150150 are not fused and the specimen is thus considered as a juvenile [17].

###### 2.1.3.1.4. *Ashoroa*.

AMP 21, a fragmentary left and a nearly complete right femur of *Ashoroa laticosta* from the late Oligocene Morawan Formation, Hokkaido, Japan, described by Inuzuka [11,13]. AMP 21 has an erupted M3 and is considered as an adult.

###### 2.1.3.1.5. Cf. *Cornwallius.*

USNM 11075 and 11076, both are right femora of *Cornwallius sookensis* from Oligocene Sooke Formation, British Columbia, Canada, described by Beatty [18]. In this study, we treated these specimens as Cf. *Cornwallius sookensis* (see discussion part of Systematic Palaeontology). USNM 11075 is missing both epiphyses and is most likely a young individual.

###### 2.1.3.1.6. *Desmostylus*.

We used three femora of *Desmostylus*. Two are UHR 18466, a nearly complete set of right and left femurs of *D. hesperus* from the Middle Miocene Uchiboro coal-bearing Formation, Sakhalin, Russia, described by Inuzuka [19]. UHR 18466 shows epiphyseal fusion in the femur and is considered an adult. The other is USNM 26134, a right femur of *D. hesperus* from Astoria Formation, Oregon, USA. USNM 26134 also shows epiphyseal fusion in the femur and is considered an adult.

##### 2.1.3.2. Potential outgroups

Femora of Perissodactyla and Proboscidea were used as potential outgroups of desmostylia. We used Grevy's zebra (*Equus grevyi*: NMMT-M43075), tapir (*Tapirus indicus*: M43531) and elephant (*Elephas maximus*: M52875). All extant specimens are adults.

### 2.2. Zircon U-Pb dating methods

**Figure 1.**
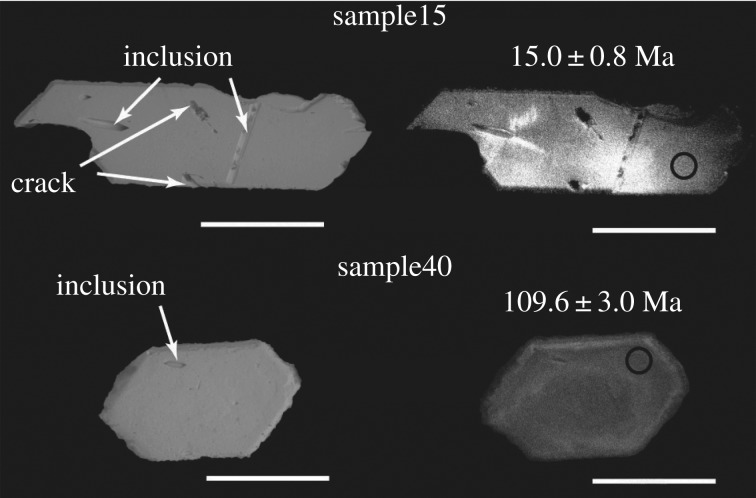
Backscattered electron (left) and cathodoluminescence (right) images from the sample. Black circles express the analysis spots and their sizes. Sample number and ^206^Pb/^238^U age are shown. Scale bar, 50 µm.

We separated host pebbly medium-grained sandstone (B110) from the EESUT-PV-0001 specimen for chronological studies. Sample B110 contains angular to subangular clasts of quartz, plagioclase, potassium feldspar, biotite, rock fragments and opaque minerals. Mineral separation was conducted at the Kyoto Fission-Track Co., Ltd, Japan. Zircons were separated from the sample (B110) using crushing, sieving, panning, magnetic separation and heavy liquid (sodium polytungstate) techniques. The separated 100 zircon grains were mounted in a PFA Teflon™ sheet and polished. To check the internal structure of zircons, they were observed in a JEOL JSML-6600LV scanning electron microscope with the cathodoluminescence (CL) and backscattered electron (BSE) detectors at the Geological Survey of Japan, AIST (Tsukuba, Japan). We selected measurement spots for the zircon U-Pb analysis using these images. The analysis spots were selected from the rim part of the zircons with no cracks or inclusions (figure 1).

Zircon U-Pb analysis was conducted with a Nu Plasma II multiple collector–ICP–mass spectrometer (MC-ICPMS; Nu Instruments, Wrexham, UK) combined with the IFRIT 260 nm femtosecond laser system (Cyber Laser Inc., Tokyo, Japan) at the Geochemical Research Center, the University of Tokyo. A baffled-type stabilizer [20] was inserted between the ablation chamber and the argon gas inlet to obtain stable isotopic ratios. Prior to the U-Pb isotopic analysis, pre-ablation procedure was adopted to eliminate possible surface contaminations of U and Pb [21]. The measurement spot size was about 15 µm in diameter. Monitored peaks were of ^202^Hg, ^204^(Hg + Pb), ^206^Pb, ^207^Pb, ^208^Pb, ^232^Th, and ^238^U. Detailed analytical conditions are shown in electronic supplementary material, table S1. The Nancy 91500 zircon (^206^Pb/^238^U age of 1062.4 ± 0.4 Ma; [22]) was used as the primary standard zircon. In addition, the OD-3 zircon (^206^Pb/^238^U age of 33.0 ± 0.1 Ma; [23]) was measured as the secondary standard sample during the measurement of unknown samples. Data were acquired in sequences of 54 analyses, consisting of three analyses of NIST (National Institute of Standards and Technology, USA) SRM 612 glass standard, three Nancy 91500 zircon, one OD-3 zircon, 14 unknown, three SRM 612 standard, and three Nancy 91500 zircon, with one gas blank before each analysis.

After the analyses, we processed the data with Microsoft Excel and Isoplot 4.15 [24] and plotted all the data on a concordia diagram. Then we chose concordant zircon data with the 2σ error-ellipse overlapping the concordia curve (a plot of ^206^Pb/^238^U (ordinate) against ^207^Pb/^235^U (abscissa) for concordant samples of various ages should define a single curve; the curve is named concordia curve and is the locus of all concordant U-Pb ages) and drew a probability density plot with a histogram (data interval of 10 Myr) using the ^238^U-^206^Pb data.

## 3. Geological setting and age

**Figure 2.**
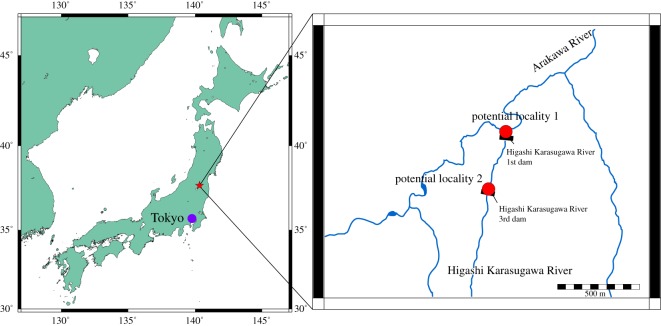
The locality map of EESUT-PV-0001.

As the EESUT-PV-0001 specimen is a historical collection, the field records are lacking. According to memories of local people, the fossil was discovered during the construction of a debris flow barrier (sabo dam) in Higashi Karasugawa River. Based on the local address on the label and interviews with local people, we speculate the exact locality to be within two possibilities, which are only 350 m apart. In either case, the exact locality is covered by the concrete foundation of the sabo dams. One possibility is in the Sabo Dam No. 3 of Higashi Karasugawa River (figure 2). This dam is located in Tate-no-koshi in the old address system, which is concordant with the label information. The other possibility is the junction between Higashi Karasugawa River and Arakawa River near the Sabo Dam No. 1 of Higashi Karasugawa River (figure 2). Details of the possible locality are provided in §5.1.

### 3.1. Geological setting

The lower to middle Miocene Tsuchiyutoge Formation [25] is distributed in the Tsuchiyutoge Area, west of Fukushima City, Fukushima Prefecture. This formation, approximately 600 m in thickness, unconformably covers pre-Neogene rocks and consists mainly of mudstone intercalated with tuff layers [25,26]. Marine fossils (such as bivalves and calcareous nannofossils) and plants were reported. These calcareous nannofossils (such as *Cylicargolithus floridanus* and *Sphenolithus heteromorphus*) indicate CN4 zone (13.5–14.9 Ma; [26,27]).

#### 3.1.1. Potential locality no. 1—the Sabo Dam No. 3 of Higashi Karasugawa River

Most of the outcrops were covered by the foundation. However, the outcrop is exposed on the left bank of the river. The sediments are composed of medium-grained volcanoclastic sandstone containing many rock fragments (figure 2).

#### 3.1.2. Potential locality no. 2—confluence point of Higashi Karasugawa River and Arakawa River

The outcrop is completely covered by concrete foundation of the sabo dam, but the upper strata with several metres in thickness are exposed above the level of the foundation. The lithofacies of the strata are massive sandstone mainly composed of volcanic ash, and coloured minerals and rock fragments are very few (figure 2).

### 3.2. Host rock of this specimen and its zircon U-Pb ages

#### 3.2.1. Host rock of this specimen

A small portion of the host rock was still attached to EESUT-PV-0001, it is medium sandstone with small gravels (2–3 mm in size). These gravels were composed of coloured minerals and rock fragments. The character of this host rock was almost corresponding to lithofacies of potential locality no. 1, but it was impossible to specify an accurate horizon only by the characteristics of host rock because lithofacies at potential locality no. 2 are impossible to observe.

#### 3.2.2. Zircon U-Pb dating result

Most of the zircons separated from the host rock of EESUT-PV-0001 were light brown colour and euhedral to subhedral, with the length of about 100–450 µm. The CL and BSE images are shown in figure 1. Most of the zircons showed weak oscillatory zoning and had no metamorphic rim.

**Figure 3.**
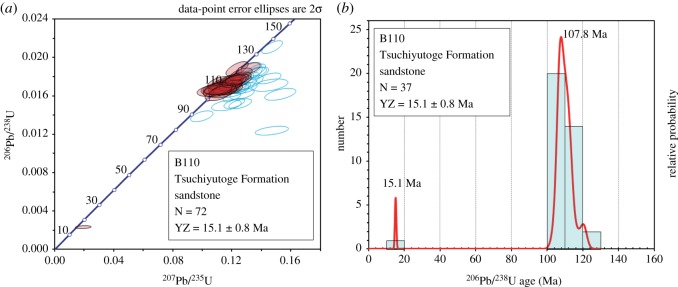
Analytical data of the sample (B110). (*a*) Concordia diagram (i.e. a plot of ^206^Pb/^238^U (ordinate) against ^207^Pb/^235^U (abscissa)) for all data; (*b*) probability density plot and histogram in the interval of 10 million years. A blue solid curve is the concordia curve, which means the locus of all concordant U-Pb ages for concordant samples of various ages that define a single curve, with open circles for the ages. Red solid circles indicate analytical data for concordant zircon grains, whereas blue open circles indicate those for discordant zircon grains. The term ‘concordant’ indicates the agreement of calculated ^238^U-^206^Pb and ^235^U-^207^Pb ages within experimental error for the same ablated portion of a zircon grain. The term ‘discordant’ indicates disagreement of calculated ^238^U-^206^Pb and ^235^U-^207^Pb ages within experimental error for the same ablated portion of a zircon grain. Discordant ages usually imply that one or both of the isotopic systems has been disturbed by some geologic event, such as metamorphism and weathering, following the initial crystallization of the zircon grain (modified from [28]). YZ: youngest zircon age. All the data to generate figure 1 are listed in electronic supplementary material, table S2.

The zircon U-Pb data from the samples are listed in electronic supplementary material, table S2. We obtained 72 analyses from 72 zircons from the sandstone attached in EESUT-PV-0001; 37 zircon data were concordant, among which 36 zircons showed Early Cretaceous (*ca* 104–120 Ma) ages (figure 3). The concordant age of the youngest zircon was 15.1 ± 0.8 Ma (2σ). The Th/U ratio of each analysis was 0.14–0.99 and fell in the range of igneous zircon, Th/U > 0.1 [29].

#### 3.2.3. Discussion about depositional age of the specimen

The sample contained many Cretaceous zircons and a Miocene zircon. The Tsuchiyutoge Formation, which the zircon samples come from, unconformably overlies the Abukuma plutonic rocks [30]. Recently, 100–118 Ma zircon U-Pb ages were obtained from the Abukuma plutonic rocks (e.g. [31]). Hence, the Early Cretaceous zircons from the sample (104–120 Ma) were presumably derived from the Abukuma plutonic rocks having the similar range of U-Pb ages. On the other hand, the youngest zircon age of 15.1 ± 0.8 Ma of the sample constrains the depositional age of the Tsuchiyutoge Formation to be 15.9 Ma or younger, considering the error, although the Miocene age was obtained only from one zircon.

## 4. Systematic palaeontology

Desmostylia Reinhart, 1953 [32]

*Emended diagnosis for Desmostylia*. Spherical femoral head, the greater trochanter and femoral head are almost the same proximal height, anteromedially flattened diaphysis, deep trochanteric fossa, well-developed lesser trochanter, clear trochanteric line. For diagnosis of other parts, see [13,16,32,33].

Paleoparadoxiidae Reinhart, 1959 [34]

Paleoparadoxiinae Reinhart, 1959 [34] *sensu* Barnes, 2013 [17]

***Paleoparadoxia*** Reinhart, 1959 [34]

*Type species*. *Paleoparadoxia tabatai*, Tokunaga, 1939 [15] (neotype NMNS PV 5601)

*Emended diagnosis for the genus*. Relatively larger femoral head with respect to the proximal width than *Neoparadoxia* and *Ashoroa*; lesser trochanter is bulged to inside; lesser angle of intertrochanteric crest than *Behemotops*, *Neoparadoxia*, *Ashoroa* and *Desmostylus*; lesser trochanter closer to femoral head than *Desmostylus*, *Cornwallius* and *Behemotops*. For diagnosis of other parts, see [2,34].

***Paleoparadoxia* sp.**

Figures 4 and 5.

**Figure 4.**
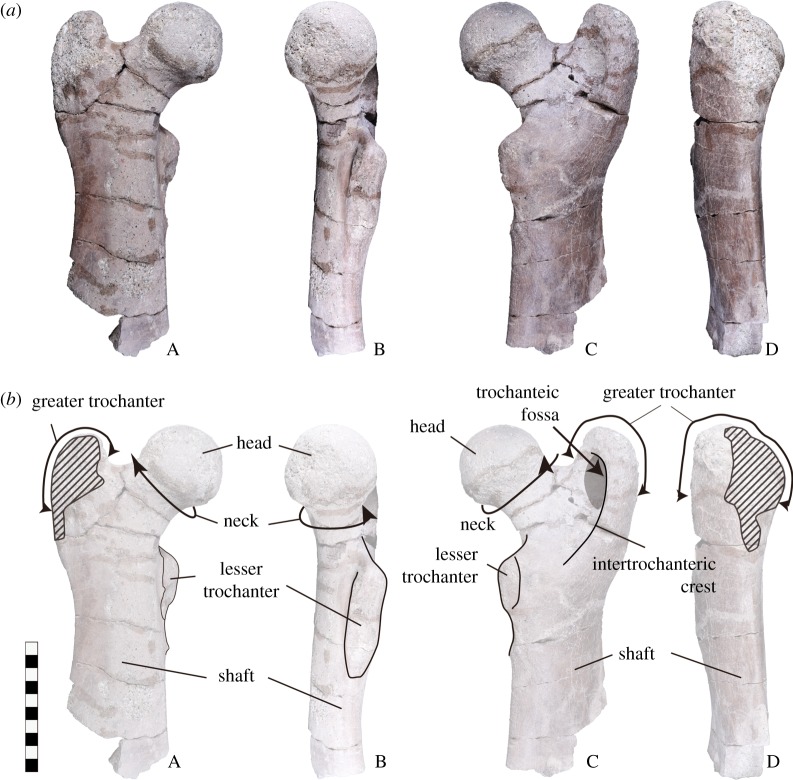
The right femur of *Paleoparadoxia* (EESUT-PV-0001). (*a*) Photograph and (*b*) line drawing. A: cranial view; B: interior view; C: caudal view; and D: exterior view. The scale bar is 10 cm.

**Figure 5.**
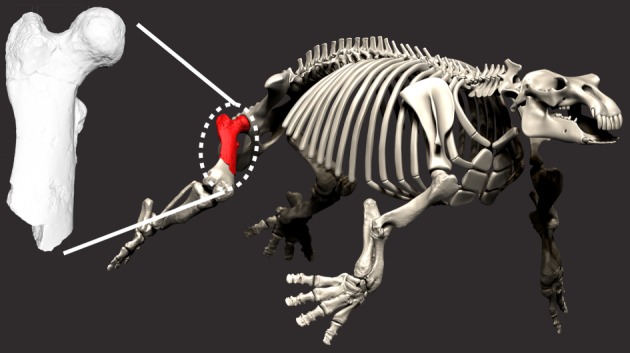
*Paleoparadoxia* skeleton and 3D model of EESUT-PV-0001.

*Material*. A right femur (EESUT-PV-0001).

*Locality*. The right bank of the Higashi Karasugawa River. The age of EESUT-PV-0001 is approximately 16–13.53 Ma by zircon and calcareous nannofossils dating.

*Comparative descriptions*. At the time of discovery, there were a lot of elements, but the only specimens still retained in collections are the single right femur and a fragment of bone (see §5). EESUT-PV-0001 is missing the distal part of the femur and a part of the greater trochanter, but the surface of EESUT-PV-0001 is well-preserved and muscle scars are well observable. Measurements are listed in table 1. The femoral head of EESUT-PV-0001 is spherical like that of most other desmostylians except for *Ashoroa* (AMP 21), which is less spherical. The femoral head of EESUT-PV-0001 (the transverse diameter (TD)/the femoral head with the maximum proximal width (MPW) = 0.53) is relatively larger with respect to the proximal width than in *Ashoroa* (AMP 21: TD/MPW = 0.45) and *Neoparadoxia* (UCMP 81302: TD/MPW = 0.47), but almost the same size as in *Behemotops* (AMP 22: TD/MPW = 0.49), *Paleoparadoxia* (NMNS-PV5601: TD/MPW = 0.50) and *Desmostylus* (UHR 18466: TD/MPW = 0.55–0.62 and USNM 26134: TD/MPW = 0.50). It is also different from the semispherical femoral heads of Perissodactyla (NSMT-M43075 and M43531) and Proboscidea (NSMT-M52875)*.* EESUT-PV-0001 does not have a fovea capitis, which differs from Perissodactyla (NSMT-M43075 and M43531) and Proboscidea (M52875). Additionally, the third trochanter of EESUT-PV-0001 does not form a process, unlike that of Perissodactyla (NSMT-M43075 and M43531).

**Table 1.** Measurements (in mm) of EESUT-KZ-0001 and comparative specimens. Measurement data are from the original data of this study and [10,19]. *Neoparadoxia cecilialina* (LACM 150150) is currently mounted in display and accurate measurements have not been taken. Therefore, we take measurements based on photographic images in ImageJ2 (Fiji) [12]. The photos were taken by Daniel N. Gabai. Cf. *Cornwallius sookensis* (USNM 11076) was cut for cross-section observation, so measurement was not carried out*.*

measuring points of the femur (mm)	EESUT-PV-0001	*Behemotops* (AMP 22)	*Paleoparadoxia* (NMNS PV-5601)	*Neoparadoxia* (UCMP 81302)	*Neoparadoxia* (LACM 150150)	*Ashoroa* (AMP 21)	Cf. *Cornwallius* (USNM 11075)	*Desmostylus* (UHR 18466 L)	*Desmostylus* (UHR 18466 R)	*Desmostylus* (USNM 26134)
maximum length	250+	459	372	507	449	267+	184	404	410	300
maximum proximal width	120	175	145	193	168+	88	—	152	142	106
length between greater and lesser trochanters	120	145	121	159	—	—	—	151	154	108
transverse diameter of head	64	86	71	91	91	39	—	84	87	53
cranio-caudal diameter of head	60	83	67	83	103	44	—	85	86	51
thickness of greater trochanter	59	92	68	85	—	49	—	76	74	49
length of femoral neck	67	95	64	98	—	—	—	108	120	68

The proximal height of the greater trochanter and femoral head of EESUT-PV-0001 is almost the same (almost coeval). This character of a coeval greater trochanter and femoral head is shared by *Ashoroa* (AMP 21), *Neoparadoxia* (UCMP 81302), *Paleoparadoxia* (NMNS-PV5601) and *Desmostylus* (UHR 18466 and USNM 26134). The thickness of the greater trochanter of EESUT-PV-0001 (thickness of greater trochanter (TGT)/the femoral head with the maximum proximal width (MPW) = 0.49) is thinner than that of *Neoparadoxia* (UCMP 81302: TGT/MPW = 0.44).

The well-developed lesser trochanter of EESUT-PV-0001 forms a distinct process as in *Paleoparadoxia* (NMNS-PV5601) and *Neoparadoxia* (UCMP 81302 and LACM 150150), whereas it does not form a distinct process in *Ashoroa* (AMP 21), *Desmostylus* (USNM 26134) and *Behemotops* (AMP 23). The lesser trochanter of EESUT-PV-0001 is situated just distal to the femoral head on the medial border, similar in condition to *Neoparadoxia* (UCMP 81302 and LACM 150150), *Paleoparadoxia* (NMNS-PV5601) and *Cornwallius* (USNM 11075), but different from *Ashoroa* (AMP 21), *Desmostylus* (UHR 18466 and USNM 26134) and *Behemotops* (AMP 23). In *Ashoroa* (AMP 21), *Desmostylus* (UHR 18466 and USNM 26134) and *Behemotops* (AMP 23) it is more posteriorly positioned than that of EESUT-PV-0001.

The angle of the intertrochanteric crest is shallower than that of *Desmostylus* (UHR 18466 and USNM 26134), *Neoparadoxia* (UCMP 81302), *Ashoroa* (AMP 21) and *Behemotops* (AMP 23). In addition, the line of the intertrochanteric crest is less developed than in *Desmostylus* (UHR 18466 and USNM 26134), *Neoparadoxia* (UCMP 81302) and *Behemotops* (AMP 23), but is about the same as in *Paleoparadoxia* (NMNS PV-5601). The diaphysis of EESUT-PV-0001 is a flattened elliptic shape in the cross-section, similar to *Paleoparadoxia* (NMNS-PV5601) and *Neoparadoxia* (UCMP 81302 and LACM 15015), but differing from *Ashoroa* (AMP 21), *Behemotops* (AMP 23) and *Desmostylus* (USNM 26134), which have a cross-sectional shape closer to a circle than EESUT-PV-0001.

The fractured surface of EESUT-PV-0001 shows a thick cortical bone and seems to lack a medullary cavity. In contrast, *Cornwallius* (USNM 11076) has a medullary cavity [18], and *Desmostylus* (GSJ F07745, 07748) has a spongy organization and lacks a medullary cavity [35]. There is no available published information of the other desmostylians, but this feature of EESUT-PV-0001 is different from *Desmostylus* (GSJ F07745, 07748) and *Cornwallius* (USNM 11075 and 11076).

*Discussions*. EESUT-PV-0001 was identified as *Paleoparadoxia* based on the characteristics described above. In addition, we propose new diagnostic characters in femoral morphology. Several previous studies included analyses of femoral characteristics of Desmostylia [10,11,14,36]. According to Inuzuka [36], the differences between *Desmostylus* and *Paleoparadoxia* (note that *Paleoparadoxia sensu* Inuzuka [36] included specimens of genus *Archaeoparadoxia*, *Paleoparadoxia* and *Neoparadoxia*) are: 1. femoral head of *Desmostylus* is larger than that of *Paleoparadoxia*, 2. femoral neck of *Paleoparadoxia* is longer and thinner than *Desmostylus*, 3. lesser trochanter and its extension of *Desmostylus* is longer than that of *Paleoparadoxia*. Inuzuka [36] only used deformed *Desmostylus* femora. In this study, we used un-deformed femora for comparisons. We found only the character 3 to be valid to distinguish *Paleoparadoxia* from *Desmostylus*. Inuzuka [14] made a simple comparison list for femora of *Behemotops*, *Paleoparadoxia* (possibly the specimens currently referred to as the holotype of *Neoparadoxia*) and *Desmostylus,* yet femoral morphology is not yet characterized for official identification at the generic level. Our study is the first report in which morphological characteristics were compared for all desmostylian genera, whose femora are known (*Behemotops*, *Paleoparadoxia*, *Neoparadoxia*, *Ashoroa*, *Cornwallius* and *Desmostylus*). EESUT-PV-0001 has the best-preserved femoral surface of all *Paleoparadoxia* specimens. In the specimen, muscle scars are easily observable, which makes the specimen useful for future studies that rely on accurate muscle maps for modelling studies of locomotion of the hind limb.

We note that some past studies did not clearly show the diagnostic characters of desmostylians (e.g. [37,38]). In these papers, diagnosis is not explicitly written, or comparative analyses are not enough. Some specimens were assigned to desmostylians heavily relying on their occurrence in a formation or its concordant age without careful assessment of the anatomical basis of their identifications. Many of them are included in databases (e.g. PBDB http://paleodb.org/), and then they are used in big data analyses [39–44]. For example, the femora described as those of *Cornwallius* [18] were primarily identified based on little evidence that its morphology is similar to those of other desmostylians but is smaller and different from *Behemotops proteus* [6], the only other desmostylian known from the synchronous age and general location at the time. Since then, evidence of at least one other smaller desmostylian, *Seuku*, has been found in the same age and region [45] suggesting that there is greater diversity than previously expected and we need more caution in making assumptions about identities of unassociated specimens. From the above, we should be more cautious in making assumptions about identities of fossil specimens unassociated with primary locality information.

## 5. Discussion

As we mentioned above, EESUT-PV-0001 was known as a ‘dinosaur’ bone by locals. This ‘dinosaur’ specimen was long-forgotten but was luckily kept with associated information written on a piece of note, and thus we were able to recover two equally possible localities even more than 60 years after the first discovery. On the note, a person's name (Tadayasu Azuma), year/date (14 June 1955) and the geographical location are written (figure 2). The geographical location reads ‘the right bank of the Higashi Karasugawa River, Tatenokoshi, Tsuchiyu Onsen Town, Fukushima City’. Thus, as a part of this research, we interviewed local people and reviewed archived documents and photos in order to specify a possible locality of the fossil and acknowledge why the specimen was brought to the University of Tsukuba.

Tsuchiyu Onsen (Hot spring) is a mountainous town famous till now for hot springs with more than 1400 years of history. This area is, however, also known for destructive flooding due to the steep slopes of the Arakawa river system (Arakawa means a tyrant river in Japanese, implying that this area has suffered from flooding for a long time). Only in the Arakawa river system, there are more than 35 sabo dams (debris flow control structures) to trap debris flow easily caused by heavy rains. Because the river system flows towards the capital city of Fukushima Prefecture, the erosion control of the region has been directly managed by the Ministry of Land, Infrastructure and Transport. As a result, documents about the historical sabo dams and photographs taken during the construction projects are well-archived in the local bureau, the Azuma Mountains Erosion Control Branch Office. According to the available documents, the labelled address corresponds to the Higashi Karasugawa River 3rd dam (figure 2), for which the construction was launched in July, 1952 and completed in March, 1953. Thus, if the piece of note truly contains the original information, the specimen must have been discovered during the construction.

Over the course of the study, we found two people who knew about the fossil and/or Mr Tadayasu Azuma. One is Mr Kazusuke Tanno, a local who worked with Mr Azuma for multiple sabo dam projects, and the other is Mr Yukio Sugeno, a retired high school science teacher. Both of them have known the fossil as a ‘dinosaur’ bone. In addition, we were also able to communicate with the wife of Mr Azuma's oldest son to get information from her husband.

**Figure 6.**
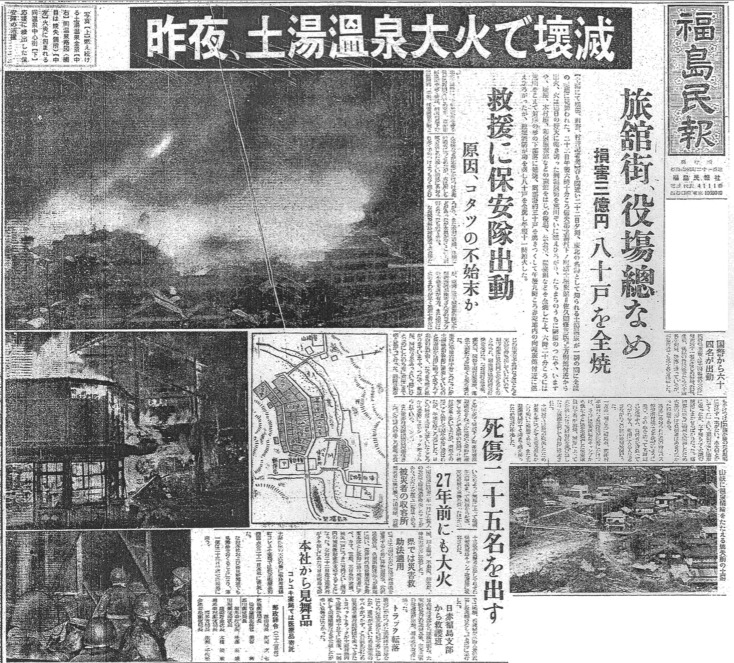
Newspaper of Tsuchiyu Onsen fire disaster.

When we showed the femur to Mr Tanno, he immediately recognized it. According to his memory, a large ‘dinosaur’ bone with a rib bone and some fragments were found by Mr Tadayasu Azuma during the construction of the Higashi Karasugawa River 1st Dam (figure 2), which is only about 350 m north of the 3rd Dam. Mr Tanno says that the fossils were discovered at the intersection between the Higashi Karasugawa River and its mainstream river, Arakawa River, while they were digging the bedrock to make steps towards the mainstream. It is said that Mr Azuma kept the fossils in his house for a while, but the ‘dinosaur’ bones were later displayed in the Village Hall. Then, at some point, a university teacher (but not Tokyo Educational University, predecessor of the University of Tsukuba) took the well-preserved ones (presumably, a femur and a rib) with him. Mr Tanno remembers it to have happened around 1954. On 22 February in the year of 1954, Tsuchiyu Onsen experienced an unforgettable devastating fire, which destroyed most of the city and caused damage valued at $2.7 million dollars (figure 6), resulting that Tsuchiyu Onsen village of Shinobu County was merged into Fukushima City as Tsuchiyu Onsen town on 1 April 1955. During the incident, the Village Hall also caught on fire. However, the small bones survived from it and were in town until a high school teacher brought them away. We further checked newspapers issued in 1954 and before, but we could not find any clue about the fossils, probably because scientific articles were not common in the newspaper at that time. However, we confirmed that the discovery of ‘dinosaur’ bones was at least known by local science teachers. Mr Sugeno heard about it in the 1960s from his colleague, Mr Minoru Katagiri, who was also a geology teacher/a geologist. Mr Tadayasu Azuma's oldest son gave us another story. According to him, he is the one who found the fossils from the 3rd Dam when he was helping his father. He also knows that the fossils belong to *Desmostylus*. Thus, some communication might have been made between a scientist and him at least until the initial identification, but the scientist did not formally report it, and the specimen was long-forgotten in a museum cabinet. Nevertheless, we need to emphasize that Mr Tadayasu Azuma's oldest son did not see the femur for the indirect interview.

As described above, there is a discrepancy in the fossil locality between Mr Tanno's memory and the note associated with the specimen. With current information, we cannot weigh either possibility due to the following reasons. First, because sedimentary facies in the outcrops of both dams are lithologically compatible with a portion of sediment which remained to be attached to the fossil, we cannot determine the specific locality based on geology. Second, based on our historical investigation, we know that the label contains geographical information after 1 April 1955, and then we must presume that the piece of the note was written with some time lag perhaps by a third person, which can result in partially incorrect information. Third, the construction year of the 1st Dam, beginning in 1947 and ending in 1951, is not contradictory to Mr Tanno's memory and the labelled information.

**Figure 7.**
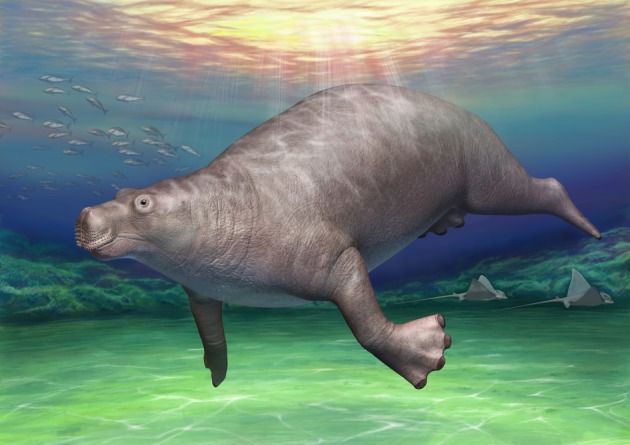
Life reconstruction of *Paleoparadoxia* from Tsuchiyu Onsen Town. This artistic image was constructed based on a combination of photogrammetric 3D models of original skeletal fossils by using PhotoScan v. 1.4.0 [46], including EESUT-PV-0001 (figure 5), and designed models of missing parts. This image gives more accurate proportion of *Paleoparadoxia* than ever reconstructed for the animal.

Although we could not identify the specific locality of EESUT-PV-0001, we were able to mark two equally possible localities, which were only 350 m apart. This could be done only because of the written record kept with the fossil in combination with local interviews. Fossils become museum specimens only when they are documented with associated records. Otherwise, they become uninformative rocks. We believe that this specimen is a good lesson not only for vertebrate palaeontologists but also for all museum curators and researchers (figure 7).

Many studies increasingly use the database like the PBDB database (http://paleodb.org/) to extract information for big data analyses of fossils. This is one of the many reasons for the importance of specimen occurrence information. In marine mammal studies, some recent examples for the use of PBDB include ecological analysis of marine mammals versus diatoms as primary producers and that of marine mammals versus other animals [39,47], the evolution of gigantism in whales and sirenians [48,49], and reconstruction of desmostylian habitats [50]. Correct provenance data (information of locality and occurrence) are requisites for these studies. It is very important to share the information of primary identification so that specimens are not forgotten like the case of this study. However, when using primary identification data and its occurrence records for research, it is very important to pay attention because there is a possibility that it will cause large change in interpretation of the result.

## 6. Conclusion

A ‘dinosaur’ femur was long-forgotten over 60 years and was discovered from the geological collection room at the national university in Japan. The morphological features of this femur correspond to those of *Paleoparadoxia*. As a result of interviews, we find two potential localities of this forgotten *Paleoparadoxia*. Combining the zircon data of the host rock and the upper limit age of the strata, the age of this ‘dinosaur’ is 16–13.53 Ma. Rediscovery of this specimen tells us about the importance of recording secondary information of museum specimens accurately, especially as it is difficult to know how informative or rare a specimen may turn out to be when examined within a comparative context that allows for their proper identification.

## Supplementary Material

Table S1

## Supplementary Material

Table S2

## Permission to carry out fieldwork

We were not required to get permission prior to conducting our fieldwork. Permission to write interview contents has been obtained from all interviewees.

## Data accessibility

Specimens are at University of Tsukuba, Tsukuba, Ibaraki, Japan. All other data is listed in figures and tables.
