# Supplementary material for: A long-forgotten ‘dinosaur’ bone from a museum cabinet, uncovered to be a Japan's iconic extinct mammal, Paleoparadoxia (Desmostylia, Mammalia)
Source: R Soc Open Sci. 2018 Jul 25;5(7):172441. doi: 10.1098/rsos.172441 (PMC6083731; doi:10.1098/rsos.172441)
Supplement: Table S1 [file rsos172441supp1.docx]

| **Laser ablation system** |  |
| --- | --- |
| Model | IFRIT Type-C (Cyber Laser Inc., Tokyo, Japan) |
| Laser type (wave length) | Ti:S femtosecond laser (THG 260 nm) |
| Laser energy | 5 J cm^-2^ |
| Pulse width | 230 fs |
| Repetition rate | 10–20 Hz |
| Crater size | 15 µm |
| Sampling mode | Single hole drilling |
| Pre-ablation time | 0.2–0.3 s |
| Ablation time | 2 s |
| Carrier gas | He gas and Ar make-up gas combined outside ablation cell |
| He gas flow rate | 0.62 L min^-1^ |
| Ar make-up gas flow rate | 0.88 L min^-1^ |
| Signal smoothing device | Baffle type [20] |
|  |  |
| **Multiple collector-ICP-MS system** |  |
| Model | Nu Plasma II MC-ICPMS (Nu instruments, Wrexham, UK) |
| Forward power | 1300 W |
| Detection system | Mixed Faraday-Daly-multiple-ion counting array |
| Forward power | [51] |
| Detectors and Monitor elements  (multipliers : IC) |  |
|  | IC0 : ^208^Pb, IC3 : ^204^(Hg + Pb), IC4 : ^202^Hg |
| (Daly detectors : D) | D1 : ^207^Pb, D2 : ^206^Pb, D5 : ^238^U |
| (Faraday cup : H) | H8 : ^232^Th |
| Integration time | 4 s |
| Data acquisition method | Time Resolved Analysis |
|  |  |
| **Standard material** |  |
| Primary standard zircon | Nancy 91500 [22] |
| Secondary standard zircon | OD-3 [23] |
| **Laser ablation system** |  |
| Model | IFRIT Type-C (Cyber Laser Inc., Tokyo, Japan) |
| Laser type (wave length) | Ti:S femtosecond laser (THG 260 nm) |
