# Supplementary material for: A long-forgotten ‘dinosaur’ bone from a museum cabinet, uncovered to be a Japan's iconic extinct mammal, Paleoparadoxia (Desmostylia, Mammalia)
Source: R Soc Open Sci. 2018 Jul 25;5(7):172441. doi: 10.1098/rsos.172441 (PMC6083731; doi:10.1098/rsos.172441)
Supplement: Table S2 [file rsos172441supp2.docx]

| Sample | Comment | Isotope ratios | | | | | | | | | | | | Age (Ma) | | | | | |  |
| --- | --- | --- | --- | --- | --- | --- | --- | --- | --- | --- | --- | --- | --- | --- | --- | --- | --- | --- | --- | --- |
|  |  | ^207^Pb/^235^U |  | 2σ | ^206^Pb/^238^U |  | 2σ | ^208^Pb/^232^Th |  | 2σ | ^207^Pb/^206^Pb |  | 2σ | ^235^U-^207^Pb |  | 2σ | ^238^U-^206^Pb |  | 2σ | Th/U |
| **1** | concordant | 0.11819 | ± | 0.00702 | 0.01729 | ± | 0.00063 | 0.005307 | ± | 0.001163 | 0.049574 | ± | 0.002332 | 113.4 | ± | 6.4 | 110.5 | ± | 4.0 | 0.52 |
| **2** | concordant | 0.11580 | ± | 0.00548 | 0.01743 | ± | 0.00062 | 0.005194 | ± | 0.001134 | 0.048187 | ± | 0.001517 | 111.3 | ± | 5.0 | 111.4 | ± | 3.9 | 0.43 |
| **3** | concordant | 0.10968 | ± | 0.00750 | 0.01677 | ± | 0.00062 | 0.005171 | ± | 0.001132 | 0.047439 | ± | 0.00273 | 105.7 | ± | 6.9 | 107.2 | ± | 3.9 | 0.86 |
| **4** | concordant | 0.11820 | ± | 0.00735 | 0.01687 | ± | 0.00062 | 0.005657 | ± | 0.001249 | 0.050827 | ± | 0.002557 | 113.4 | ± | 6.7 | 107.8 | ± | 3.9 | 0.27 |
| **5** | concordant | 0.11194 | ± | 0.00628 | 0.01717 | ± | 0.00062 | 0.005252 | ± | 0.001148 | 0.047288 | ± | 0.002038 | 107.7 | ± | 5.7 | 109.7 | ± | 3.9 | 0.62 |
| **6** | concordant | 0.11567 | ± | 0.00566 | 0.01726 | ± | 0.00061 | 0.00519 | ± | 0.001134 | 0.048616 | ± | 0.001636 | 111.1 | ± | 5.1 | 110.3 | ± | 3.9 | 0.42 |
| **7** | discordant | 0.13298 | ± | 0.00629 | 0.01822 | ± | 0.00065 | 0.005754 | ± | 0.001254 | 0.052945 | ± | 0.001661 | 126.8 | ± | 5.6 | 116.4 | ± | 4.1 | 0.99 |
| **8** | concordant | 0.11966 | ± | 0.00604 | 0.01734 | ± | 0.00062 | 0.005049 | ± | 0.001102 | 0.050052 | ± | 0.001791 | 114.8 | ± | 5.5 | 110.8 | ± | 3.9 | 0.76 |
| **9** | concordant | 0.11903 | ± | 0.00581 | 0.01743 | ± | 0.00062 | 0.005292 | ± | 0.001156 | 0.049534 | ± | 0.001657 | 114.2 | ± | 5.3 | 111.4 | ± | 3.9 | 0.47 |
| **10** | concordant | 0.11090 | ± | 0.00585 | 0.01696 | ± | 0.00061 | 0.00507 | ± | 0.001107 | 0.047437 | ± | 0.001839 | 106.8 | ± | 5.3 | 108.4 | ± | 3.8 | 0.64 |
| **11** | discordant | 0.13496 | ± | 0.00665 | 0.01851 | ± | 0.00066 | 0.005925 | ± | 0.001294 | 0.052889 | ± | 0.001804 | 128.5 | ± | 5.9 | 118.2 | ± | 4.2 | 0.54 |
| **12** | discordant | 0.13215 | ± | 0.00757 | 0.01739 | ± | 0.00063 | 0.005989 | ± | 0.001312 | 0.05513 | ± | 0.002444 | 126.0 | ± | 6.8 | 111.1 | ± | 4.0 | 0.46 |
| **13** | discordant | 0.12177 | ± | 0.00572 | 0.01753 | ± | 0.00062 | 0.005534 | ± | 0.001207 | 0.050378 | ± | 0.001558 | 116.7 | ± | 5.2 | 112.0 | ± | 3.9 | 0.67 |
| **14** | concordant | 0.11489 | ± | 0.00567 | 0.01702 | ± | 0.00060 | 0.005139 | ± | 0.001121 | 0.048954 | ± | 0.001676 | 110.4 | ± | 5.2 | 108.8 | ± | 3.8 | 0.73 |
| **15** | concordant | 0.01915 | ± | 0.00442 | 0.00234 | ± | 0.00012 | 0.001039 | ± | 0.000299 | 0.059401 | ± | 0.01333 | 19.3 | ± | 4.4 | 15.1 | ± | 0.8 | 0.61 |
| **16** | discordant | 0.12449 | ± | 0.00599 | 0.01676 | ± | 0.00043 | 0.004564 | ± | 0.001248 | 0.053866 | ± | 0.002187 | 119.1 | ± | 5.4 | 107.2 | ± | 2.8 | 0.43 |
| **17** | discordant | 0.11996 | ± | 0.00494 | 0.01586 | ± | 0.00040 | 0.003972 | ± | 0.001084 | 0.054876 | ± | 0.001787 | 115.0 | ± | 4.5 | 101.4 | ± | 2.5 | 0.49 |
| **18** | discordant | 0.12076 | ± | 0.00872 | 0.01653 | ± | 0.00058 | 0.005616 | ± | 0.003119 | 0.052991 | ± | 0.003351 | 115.8 | ± | 7.9 | 105.7 | ± | 3.6 | 0.30 |
| **19** | concordant | 0.11976 | ± | 0.00684 | 0.01751 | ± | 0.00058 | 0.005363 | ± | 0.002975 | 0.049618 | ± | 0.002306 | 114.9 | ± | 6.2 | 111.9 | ± | 3.7 | 0.26 |
| **20** | concordant | 0.11422 | ± | 0.00767 | 0.01681 | ± | 0.00057 | 0.004786 | ± | 0.002656 | 0.049286 | ± | 0.002851 | 109.8 | ± | 7.0 | 107.5 | ± | 3.6 | 0.39 |
| **21** | concordant | 0.11851 | ± | 0.00697 | 0.01727 | ± | 0.00058 | 0.004922 | ± | 0.002729 | 0.049766 | ± | 0.002411 | 113.7 | ± | 6.3 | 110.4 | ± | 3.7 | 0.54 |
| **22** | discordant | 0.12289 | ± | 0.00613 | 0.01740 | ± | 0.00057 | 0.002789 | ± | 0.001546 | 0.051213 | ± | 0.001934 | 117.7 | ± | 5.5 | 111.2 | ± | 3.6 | 0.36 |
| **23** | discordant | 0.09961 | ± | 0.00627 | 0.01386 | ± | 0.00047 | 0.003706 | ± | 0.002055 | 0.052125 | ± | 0.002768 | 96.4 | ± | 5.8 | 88.7 | ± | 3.0 | 0.55 |
| **24** | concordant | 0.11089 | ± | 0.00738 | 0.01620 | ± | 0.00055 | 0.00404 | ± | 0.002242 | 0.049636 | ± | 0.002838 | 106.8 | ± | 6.7 | 103.6 | ± | 3.5 | 0.41 |
| **25** | discordant | 0.13191 | ± | 0.00670 | 0.01872 | ± | 0.00061 | 0.005506 | ± | 0.003051 | 0.051101 | ± | 0.001987 | 125.8 | ± | 6.0 | 119.6 | ± | 3.9 | 0.57 |
| **26** | discordant | 0.12941 | ± | 0.00739 | 0.01777 | ± | 0.00059 | 0.005573 | ± | 0.003092 | 0.052823 | ± | 0.002452 | 123.6 | ± | 6.6 | 113.5 | ± | 3.7 | 0.23 |
| **27** | concordant | 0.12363 | ± | 0.00617 | 0.01885 | ± | 0.00061 | 0.005084 | ± | 0.002817 | 0.047573 | ± | 0.001799 | 118.4 | ± | 5.6 | 120.4 | ± | 3.9 | 0.58 |
| **28** | discordant | 0.12664 | ± | 0.00820 | 0.01726 | ± | 0.00059 | 0.004603 | ± | 0.002553 | 0.053231 | ± | 0.002933 | 121.1 | ± | 7.4 | 110.3 | ± | 3.7 | 0.45 |
| **29** | discordant | 0.12605 | ± | 0.00726 | 0.01769 | ± | 0.00059 | 0.005184 | ± | 0.002874 | 0.051684 | ± | 0.002427 | 120.5 | ± | 6.5 | 113.0 | ± | 3.7 | 0.42 |
| **30** | concordant | 0.12366 | ± | 0.00785 | 0.01757 | ± | 0.00059 | 0.00481 | ± | 0.002669 | 0.051061 | ± | 0.002741 | 118.4 | ± | 7.1 | 112.2 | ± | 3.8 | 0.35 |
| **31** | concordant | 0.12286 | ± | 0.00713 | 0.01763 | ± | 0.00059 | 0.005525 | ± | 0.003065 | 0.050528 | ± | 0.002402 | 117.7 | ± | 6.4 | 112.7 | ± | 3.7 | 0.26 |
| **32** | concordant | 0.10783 | ± | 0.00702 | 0.01679 | ± | 0.00047 | 0.00522 | ± | 0.001552 | 0.046582 | ± | 0.002739 | 104.0 | ± | 6.4 | 107.3 | ± | 3.0 | 0.62 |
| **33** | discordant | 0.12707 | ± | 0.00674 | 0.01620 | ± | 0.00044 | 0.006079 | ± | 0.001808 | 0.056899 | ± | 0.002598 | 121.5 | ± | 6.1 | 103.6 | ± | 2.8 | 0.33 |
| **34** | concordant | 0.11537 | ± | 0.00681 | 0.01663 | ± | 0.00046 | 0.004999 | ± | 0.001487 | 0.050314 | ± | 0.00263 | 110.9 | ± | 6.2 | 106.3 | ± | 2.9 | 0.49 |
| **35** | discordant | 0.13609 | ± | 0.00603 | 0.01839 | ± | 0.00048 | 0.006013 | ± | 0.001785 | 0.053684 | ± | 0.001928 | 129.6 | ± | 5.4 | 117.4 | ± | 3.0 | 0.37 |
| **36** | discordant | 0.14726 | ± | 0.00918 | 0.01238 | ± | 0.00036 | 0.007172 | ± | 0.002137 | 0.086289 | ± | 0.00474 | 139.5 | ± | 8.1 | 79.3 | ± | 2.3 | 0.33 |
| **37** | discordant | 0.31791 | ± | 0.02086 | 0.02290 | ± | 0.00070 | 0.013624 | ± | 0.004065 | 0.100703 | ± | 0.00584 | 280.3 | ± | 16.1 | 145.9 | ± | 4.4 | 0.37 |
| **38** | discordant | 0.14500 | ± | 0.00675 | 0.01780 | ± | 0.00047 | 0.006159 | ± | 0.001829 | 0.059082 | ± | 0.002267 | 137.5 | ± | 6.0 | 113.7 | ± | 3.0 | 0.37 |
| **39** | concordant | 0.11778 | ± | 0.00798 | 0.01675 | ± | 0.00048 | 0.005359 | ± | 0.001603 | 0.050993 | ± | 0.003133 | 113.1 | ± | 7.2 | 107.1 | ± | 3.0 | 0.31 |
| **40** | concordant | 0.11820 | ± | 0.00693 | 0.01715 | ± | 0.00047 | 0.005155 | ± | 0.001535 | 0.049996 | ± | 0.002595 | 113.4 | ± | 6.3 | 109.6 | ± | 3.0 | 0.37 |
| **41** | concordant | 0.10825 | ± | 0.00638 | 0.01684 | ± | 0.00046 | 0.004959 | ± | 0.001473 | 0.046618 | ± | 0.002436 | 104.4 | ± | 5.8 | 107.7 | ± | 2.9 | 0.62 |
| **42** | concordant | 0.10656 | ± | 0.00836 | 0.01659 | ± | 0.00049 | 0.005305 | ± | 0.001586 | 0.046588 | ± | 0.003389 | 102.8 | ± | 7.7 | 106.1 | ± | 3.1 | 0.42 |
| **43** | discordant | 0.27255 | ± | 0.01245 | 0.01886 | ± | 0.00051 | 0.005508 | ± | 0.001634 | 0.104808 | ± | 0.003855 | 244.7 | ± | 9.9 | 120.4 | ± | 3.2 | 0.85 |
| **44** | concordant | 0.11652 | ± | 0.00721 | 0.01680 | ± | 0.00047 | 0.004966 | ± | 0.001478 | 0.050293 | ± | 0.002782 | 111.9 | ± | 6.6 | 107.4 | ± | 3.0 | 0.51 |
| **45** | discordant | 0.12706 | ± | 0.00817 | 0.01681 | ± | 0.00047 | 0.005721 | ± | 0.001708 | 0.054833 | ± | 0.003169 | 121.5 | ± | 7.4 | 107.4 | ± | 3.0 | 0.34 |
| **46** | discordant | 0.20215 | ± | 0.01273 | 0.01646 | ± | 0.00051 | 0.007717 | ± | 0.002133 | 0.089094 | ± | 0.004894 | 186.9 | ± | 10.8 | 105.2 | ± | 3.2 | 0.38 |
| **47** | discordant | 0.12184 | ± | 0.00576 | 0.01524 | ± | 0.00042 | 0.005898 | ± | 0.001624 | 0.057988 | ± | 0.002223 | 116.7 | ± | 5.2 | 97.5 | ± | 2.7 | 0.24 |
| **48** | discordant | 0.12388 | ± | 0.00569 | 0.01767 | ± | 0.00048 | 0.006001 | ± | 0.001648 | 0.050831 | ± | 0.001877 | 118.6 | ± | 5.1 | 112.9 | ± | 3.1 | 0.44 |
| **49** | concordant | 0.11487 | ± | 0.00637 | 0.01681 | ± | 0.00047 | 0.00577 | ± | 0.001594 | 0.049577 | ± | 0.002367 | 110.4 | ± | 5.8 | 107.4 | ± | 3.0 | 0.23 |
| **50** | concordant | 0.13112 | ± | 0.00655 | 0.01886 | ± | 0.00052 | 0.0072 | ± | 0.00198 | 0.050425 | ± | 0.002095 | 125.1 | ± | 5.9 | 120.4 | ± | 3.3 | 0.33 |
| **51** | discordant | 0.14741 | ± | 0.00575 | 0.02108 | ± | 0.00056 | 0.007494 | ± | 0.002055 | 0.05071 | ± | 0.001438 | 139.6 | ± | 5.1 | 134.5 | ± | 3.6 | 0.38 |
| **52** | discordant | 0.14582 | ± | 0.01005 | 0.01711 | ± | 0.00052 | 0.003368 | ± | 0.000934 | 0.0618 | ± | 0.003822 | 138.2 | ± | 8.9 | 109.4 | ± | 3.3 | 0.59 |
| **53** | discordant | 0.11986 | ± | 0.00507 | 0.01690 | ± | 0.00046 | 0.005692 | ± | 0.001566 | 0.051453 | ± | 0.001672 | 114.9 | ± | 4.6 | 108.0 | ± | 2.9 | 0.19 |
| **54** | concordant | 0.11951 | ± | 0.00617 | 0.01772 | ± | 0.00049 | 0.006393 | ± | 0.001759 | 0.048921 | ± | 0.002129 | 114.6 | ± | 5.6 | 113.2 | ± | 3.1 | 0.33 |
| **55** | discordant | 0.19727 | ± | 0.01663 | 0.01877 | ± | 0.00063 | 0.016649 | ± | 0.004667 | 0.076207 | ± | 0.005886 | 182.8 | ± | 14.1 | 119.9 | ± | 4.0 | 0.14 |
| **56** | discordant | 0.12237 | ± | 0.00613 | 0.01680 | ± | 0.00047 | 0.005618 | ± | 0.001544 | 0.052835 | ± | 0.0022 | 117.2 | ± | 5.5 | 107.4 | ± | 3.0 | 0.42 |
| **57** | discordant | 0.18654 | ± | 0.01086 | 0.01660 | ± | 0.00049 | 0.005787 | ± | 0.001595 | 0.081504 | ± | 0.00408 | 173.7 | ± | 9.3 | 106.1 | ± | 3.1 | 0.53 |
| **58** | discordant | 0.12582 | ± | 0.00632 | 0.01518 | ± | 0.00043 | 0.00487 | ± | 0.001341 | 0.060117 | ± | 0.002508 | 120.3 | ± | 5.7 | 97.1 | ± | 2.7 | 0.34 |
| **59** | concordant | 0.11939 | ± | 0.00657 | 0.01713 | ± | 0.00048 | 0.006083 | ± | 0.001674 | 0.050534 | ± | 0.002386 | 114.5 | ± | 6.0 | 109.5 | ± | 3.1 | 0.40 |
| **60** | concordant | 0.11223 | ± | 0.00717 | 0.01739 | ± | 0.00053 | 0.006284 | ± | 0.000386 | 0.046805 | ± | 0.002625 | 108.0 | ± | 6.5 | 111.1 | ± | 3.4 | 0.38 |
| **61** | concordant | 0.11424 | ± | 0.00655 | 0.01674 | ± | 0.00050 | 0.006259 | ± | 0.000364 | 0.049505 | ± | 0.002419 | 109.8 | ± | 6.0 | 107.0 | ± | 3.2 | 0.50 |
| **62** | concordant | 0.11013 | ± | 0.00797 | 0.01660 | ± | 0.00052 | 0.00614 | ± | 0.000386 | 0.048123 | ± | 0.003134 | 106.1 | ± | 7.3 | 106.1 | ± | 3.3 | 0.47 |
| **63** | concordant | 0.12611 | ± | 0.00632 | 0.01831 | ± | 0.00054 | 0.006508 | ± | 0.00038 | 0.049962 | ± | 0.00203 | 120.6 | ± | 5.7 | 116.9 | ± | 3.4 | 0.30 |
| **64** | discordant | 0.12157 | ± | 0.00740 | 0.01703 | ± | 0.00052 | 0.006117 | ± | 0.000385 | 0.051767 | ± | 0.002726 | 116.5 | ± | 6.7 | 108.9 | ± | 3.3 | 0.31 |
| **65** | discordant | 0.15292 | ± | 0.00860 | 0.01616 | ± | 0.00049 | 0.007958 | ± | 0.000481 | 0.06864 | ± | 0.003247 | 144.5 | ± | 7.6 | 103.3 | ± | 3.1 | 0.30 |
| **66** | concordant | 0.11111 | ± | 0.00789 | 0.01679 | ± | 0.00053 | 0.006386 | ± | 0.00043 | 0.047993 | ± | 0.003056 | 107.0 | ± | 7.2 | 107.3 | ± | 3.3 | 0.28 |
| **67** | discordant | 0.12253 | ± | 0.00830 | 0.01687 | ± | 0.00053 | 0.005931 | ± | 0.000423 | 0.052685 | ± | 0.003165 | 117.4 | ± | 7.5 | 107.8 | ± | 3.3 | 0.22 |
| **68** | concordant | 0.12430 | ± | 0.00710 | 0.01786 | ± | 0.00054 | 0.00663 | ± | 0.000393 | 0.050489 | ± | 0.002455 | 119.0 | ± | 6.4 | 114.1 | ± | 3.4 | 0.40 |
| **69** | discordant | 0.13669 | ± | 0.00671 | 0.01834 | ± | 0.00054 | 0.004728 | ± | 0.000287 | 0.054047 | ± | 0.00213 | 130.1 | ± | 6.0 | 117.2 | ± | 3.4 | 0.26 |
| **70** | discordant | 0.11646 | ± | 0.00827 | 0.01502 | ± | 0.00048 | 0.005178 | ± | 0.000335 | 0.056234 | ± | 0.003573 | 111.9 | ± | 7.5 | 96.1 | ± | 3.0 | 0.44 |
| **71** | concordant | 0.12146 | ± | 0.00671 | 0.01759 | ± | 0.00052 | 0.006275 | ± | 0.000367 | 0.050082 | ± | 0.002329 | 116.4 | ± | 6.1 | 112.4 | ± | 3.3 | 0.44 |
| **72** | concordant | 0.11395 | ± | 0.00763 | 0.01648 | ± | 0.00051 | 0.006457 | ± | 0.000392 | 0.050145 | ± | 0.002974 | 109.6 | ± | 7.0 | 105.4 | ± | 3.2 | 0.49 |
